# Case Report: Genomic characterization of a rare skull-base plasmacytoma

**DOI:** 10.3389/fonc.2025.1645502

**Published:** 2025-11-25

**Authors:** Hasan Alanya, Sreekar Kasturi, Kanat Yalcin, Batur Gultekin, Deepika Kumar, Natalia Samuel, Diego Samper Figuera, Ketu Mishra-Gorur, Murat Gunel, E. Zeynep Erson-Omay, S. Bulent Omay

**Affiliations:** 1 Department of Neurosurgery, Yale School of Medicine, New Haven, CT, United States; 2 Department of Pathology, Yale School of Medicine, New Haven, CT, United States; 3 Department of Biomedical Informatics and Data Science, Yale School of Medicine, New Haven, CT, United States; 4 Department of Surgery, Division of Otolaryngology, Yale School of Medicine, New Haven, CT, United States

**Keywords:** skull-base plasmacytoma, genomic profiling, multiple myeloma, solitary plasmacytoma, ARID1A, KMT2D

## Abstract

Skull-base plasmacytoma (SBP) is a rare plasma cell neoplasm that typically presents as a slow-growing skull-base lesion in older adults; however, its molecular underpinnings are poorly characterized, limiting targeted therapeutic interventions and precise prognostication. We describe the case of a 67-year-old female who presented with progressive headaches, whose imaging and biopsy confirmed a solitary SBP with no evidence of systemic disease. Whole-exome sequencing revealed an elevated somatic copy number alteration burden and pathogenic variants in *ARID1A*, *KMT2D*, *BCL7A*, *PTPN11*, and *NUP214*, suggesting disruptions in chromatin remodeling, B-cell neoplasm pathogenesis, and oncogenic signaling driving the tumorigenesis. These findings provide novel insights into molecular landscape of SBP, highlighting potential for risk stratification and targeted therapy development. The case underscores the importance of comprehensive genomic profiling in rare skull-based tumors to enhance our understanding of their biology and to guide personalized clinical management.

## Introduction

Skull-base plasmacytoma (SBP) is an uncommon subset of plasma cell neoplasms that accounts for fewer than 10% of all plasmacytomas, typically presenting in patients in their sixth decade of life (1). Arising from the skull base, these lesions can pose distinctive diagnostic and therapeutic challenges due to their proximity to critical neurovascular structures. While plasmacytomas frequently originating within the bone marrow or manifest extramedullary at other anatomical sites are characterized better, SBP’s molecular characteristics remains insufficiently characterized.

Current genomic insights into plasma cell neoplasms are primarily derived from studies of multiple myeloma (MM). In MM, comprehensive profiling has identified key molecular hallmarks, including immunoglobulin heavy chain (IgH) translocations, hyperdiploidy, and recurrent mutations in genes involved in cell-cycle regulation (e.g., CCND1), RAS/RAF signaling (e.g., KRAS, NRAS, BRAF), and chromatin remodeling (e.g., ARID1A, KMT2D) (2, 3). Alterations in tumor suppressor genes such as TP53 and NF-κB pathway genes have also been implicated in disease pathogenesis (4). However, large-scale genomic studies specifically focused on skull base plasmacytomas are lacking, largely due to their rarity. Consequently, much of our current understanding has been inferred from broader investigations of multiple myeloma or solitary plasmacytomas at other anatomical sites.

Nevertheless, early lines of evidence imply that plasmacytomas—whether solitary or occurring in conjunction with MM—may harbor overlapping genetic features. In particular, extramedullary plasmacytomas outside the skull base have been shown to harbor mutations in genes similarly implicated in MM, including regulators of epigenetic modification, B-cell differentiation, and oncogenic signaling. A case report by Stawarz et al. (5) highlighted an uncommon presentation of extramedullary plasmacytoma in the absence of concurrent MM, underlining the variable clinical trajectories these neoplasms may follow. Notably, recent reports have identified recurrent alterations in *PTPN11*, *NUP214*, and *BCL7A*, pointing to a convergence on signaling pathways critical for plasma cell malignancy and underscoring the need for dedicated genomic investigations of SBP (3, 6–8).

Revealing the genetic and molecular landscape of SBP is imperative not only for improving diagnostic precision and informing treatment decisions but also for clarifying how these lesions fit within the broader spectrum of plasma cell neoplasms. Given SBP’s relative rarity, each additional genomic study enriches the existing literature and may reveal novel driver events, prognostic markers, or therapeutic vulnerabilities. In this report, we expand upon the limited body of knowledge by presenting comprehensive genomic data from a solitary SBP.

Skull-base plasmacytoma occupies a clinical and biological interface with multiple myeloma (MM). Solitary plasmacytomas account for <10% of plasma-cell neoplasms, and skull-base involvement represents a rare subset with unique anatomic constraints (1, 9). Approximately half of solitary bone plasmacytomas progress to MM within several years (10, 11), which shapes management emphasizing systemic exclusion, local control (RT), and structured surveillance. These epidemiologic ties also justify genomic profiling in SBP to identify risk features such as 1q gain and complex CNA patterns associated with poor outcomes in MM (12–14).

## Case description

A 67-year-old woman presented with new-onset posterior neck and skull pain, with imaging revealing a large expansile neoplastic mass. Her past medical history was significant for diagnosis of breast carcinoma associated with a germline frameshift deletion in the *ATM* gene, treated with a right breast total mastectomy. Additionally, she has managed conditions of asthma, hypertension controlled with clonidine, and hypothyroidism. On examination, she was alert and oriented, neurologically non-focal.

**Figure 1:**
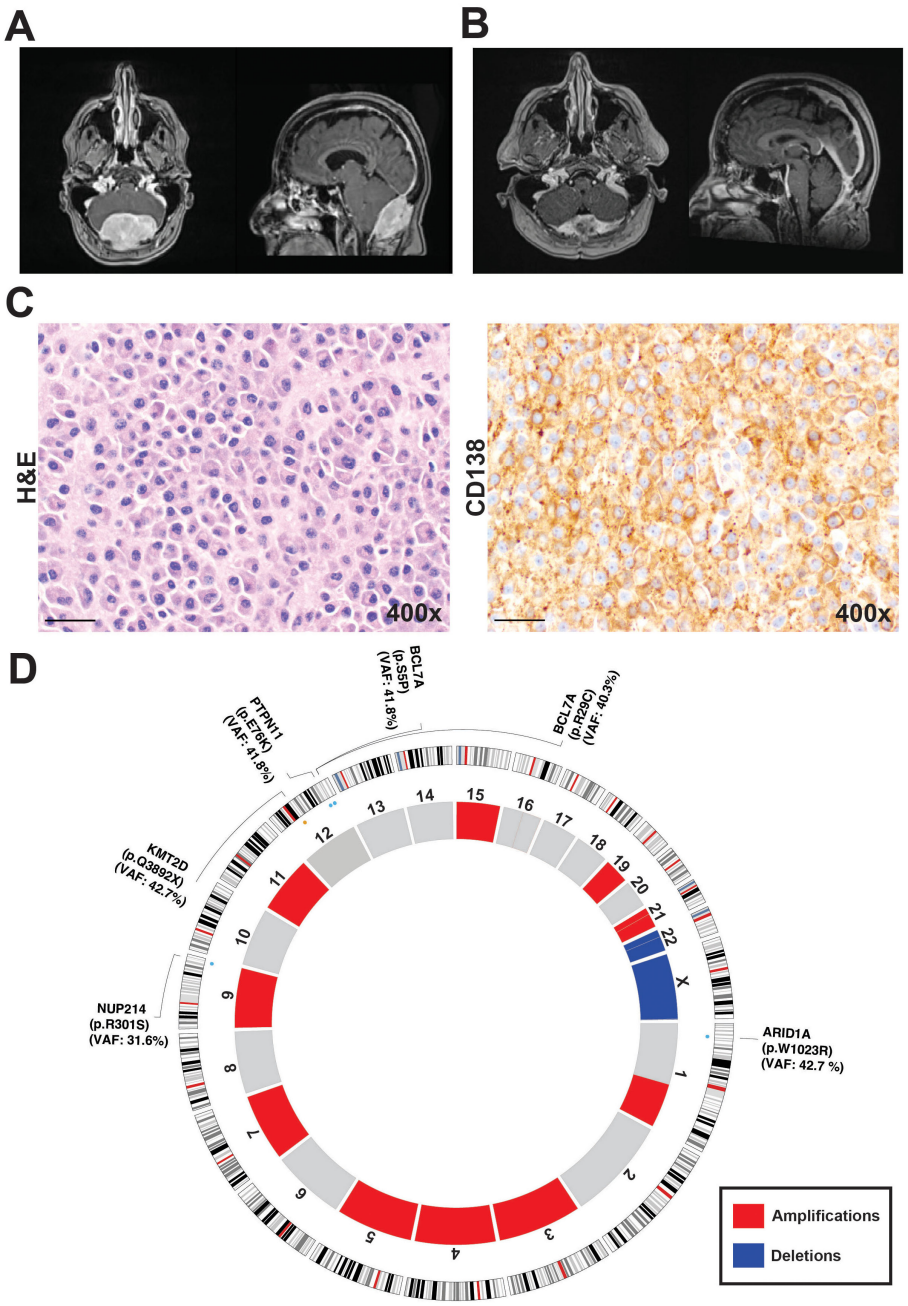
Radiological, histological and genomic characterization of the skull-based plasmacytoma case. **(A)** Preoperative magnetic resonance imaging (MRI). **(B)** MRI following initial radiation treatment following the biopsy. **(C)** 400× magnification revealing diffuse sheets of atypical plasma cells. Immunohistochemical staining for CD138, confirming plasma cell lineage and highlighting the neoplastic cells. **(D)** Circos plot generated from whole-exome sequencing data, depicting somatic copy number alterations and variants in ARID1A, KMT2D, BCL7A, PTPN11, and NUP214. Amplifications are shown in red and include regions on chromosomes 1q, 3, 4, 5, 7, 9, 11, 15, 19, and 21. Deletions, shown in blue, were identified on chromosome 22 and the X chromosome.

Magnetic resonance imaging (MRI) of her brain revealed an expansile mass in the suboccipital calvarium, measuring 6 cm craniocaudal by 4 cm anteroposterior by 7.5 cm transverse, with both intracranial and extracranial extensions (**Figure 1A**). There was also significant mass effect on the cerebellum, but with no evidence of cerebellar herniation or intraparenchymal involvement, indicating an indolent process rather than aggressive growth. The differential diagnosis was considered to include metastatic disease, hemangiopericytoma, meningioma, or plasmacytoma, given the lesion’s expansive nature and involvement of the suboccipital calvarium and adjacent dural sinuses. A suboccipital biopsy was performed to obtain a tissue-based diagnosis of the expansive mass.

Pathologic review of the biopsy specimen revealed diffuse sheets of atypical plasma cells with occasional Dutcher bodies, highlighted by CD138 staining and showing kappa light chain restriction. The Ki-67 proliferation index was approximately 5-10% and the immunohistochemical staining results showed variable CD38 expression with negative CD117 and positive CD56 expression among the plasma cells. Further immunohistochemical stains showed plasma cells positive for CD138 and kappa light chains, with negative results for CD117, CD19, CD20, and S100 (**Figure 1C**). A subset of plasma cells was positive for EMA. Together these findings were consistent with SBP. She underwent local radiotherapy of 3000 cGy in 15 fractions, followed by three cycles of Dara-VRD, which have reduced the size of the plasmacytoma and alleviated mass effect symptoms (**Figure 1B**).

At last follow-up (~16 months from diagnosis), the patient reports sustained resolution of posterior skull pain, is neurologically non-focal, and maintains independent activities of daily living. Follow up MRIs continues to demonstrate interval reduction in lesion dimensions with decreased mass effect (see **Figure 1B**) and no new enhancing disease. She transitioned to Maintenance Dara/Rev (lenalidomine) and is in serologic and clinical remission. For surveillance, serologic and biochemical monitoring and MRI of the brain every 3–4 months and bone marrow assessment is planned. PET/CT whole body is planned in at 20 months or earlier if new cytopenias, biochemical progression, or radiographic change emerges (**Table 1**). This reflects the risk of progression from solitary lesions to MM and the patient’s initial high CNA burden with 1q gain.

**Table 1:** Timeline of presentation, diagnostics, treatment, and follow-up.

Date	Event	Details
Month −3 to 0	Symptom onset	Progressive posterior neck/skull pain
Day 0	Initial MRI	6×4×7.5 cm suboccipital calvarial mass with intra/extra-cranial extension
+1 week	Biopsy	Plasma-cell neoplasm; CD138+, kappa-restricted; Ki-67 ~5–10%
+2 weeks	Systemic work-up	No marrow or systemic MM; baseline labs (SPEP/IFE/FLC) negative/polyclonal
+3 to +5 weeks	Radiotherapy	30 Gy in 15 fractions
+7 weeks	Post-RT MRI	Interval size reduction, mass-effect improved
+8 weeks onward	Systemic therapy	Dara-VRd ×8 cycles
+10 weeks onward	Systemic therapy	Dara-Rev Maintanence
+16 months	Status	Neurologically intact; clinical and serologic remission
*+20 months*	*Bone marrow assessment and* PET/CT whole body	Planned other Surveillence
Every 3 mo	Surveillance labs	Serologic monitoring, CBC, CMP
Every 3–4 mo (years 0–2)	MRI skull base	Radiographic monitoring

### Genomic analysis

Under an institutional review board-approved protocol, whole-exome sequencing (WES) was performed on tumor biopsy tissue and matched normal blood. A rigorous bioinformatic filtering strategy identified multiple somatic alterations, pointing to a genomically unstable neoplasm. From the refined variant set, five potentially known pathogenic or predicted to be deleterious somatic mutations emerged: *ARID1A* (NM_006015:p.W1023R, pathogenic), *KMT2D* (NM_003482:p.Q3892X), *BCL7A* (NM_020993:p.R29C, NM_020993:p.S5P), *PTPN11* (NM_002834:p.E76K), and *NUP214* (NM_001318324:p.R301S) (**Figure 1D**). These genes have been implicated in various tumorigenic processes, including epigenetic regulation, chromatin remodeling, B-cell differentiation, and oncogenic signaling.

Analysis of somatic copy number variants (CNVs) revealed a high overall burden of copy number changes, reflecting extensive genomic instability impacting 44.7% of the whole genome. Amplifications were detected on chromosomes 1q, 3, 4, 5, 7, 9, 11, 15, 19, and 21, while deletions were observed on chromosome 22 and the X. Gains in 1q are particularly noteworthy, as this alteration has been associated with poor outcomes in multiple myeloma and may signal a similarly aggressive biological phenotype in SBP (12, 14, 15). Moreover, increased copy number and structural changes are common features of multiple myelomas (13).

Taken together, the integrative genomic data show that the tumor harbors several putative driver point mutations and a high burden of CNVs. The cooperation of epigenetic regulators (*ARID1A*, *KMT2D*), B-cell neoplasm-associated genes (*BCL7A*), and oncogenic signaling components (*PTPN11*, *NUP214*) likely contributes to its malignant behavior together with increased genomic instability. These molecular insights shed new light on the pathogenesis of SBP and open potential avenues for targeted therapeutic interventions, particularly those focusing on epigenetic pathways, signal transduction, and DNA repair mechanisms.

Germline variant analysis confirmed the pathogenic germline variant in the ATM gene (ATM serine/threonine kinase) located on chromosome 11 (hg19: 11:108121593), characterized by a frameshift deletion (ATM: NM_000051:exon10:c.1402_1403del:p.K468Efs*18). This germline alteration has been previously associated with “Hereditary_cancer-predisposing_syndrome” in ClinVar.

### Patience perspective

The patient had a long history of migraines that gradually changed in character, becoming more intense and persistent. This prompted neuroimaging, which revealed a large skull-base mass later diagnosed as a plasmacytoma. The diagnosis was especially distressing given a prior recurrence of breast cancer one year earlier. The patient underwent biopsy, radiation, and systemic therapy with close multidisciplinary guidance and shared decision-making throughout the course of care. Following treatment, headache symptoms markedly improved, and interval imaging demonstrated significant tumor reduction. The patient subsequently achieved a complete response to induction therapy and remains on monthly maintenance treatment for multiple myeloma, with regular MRI, PET scans, bone marrow biopsies, and laboratory monitoring. The patient expressed appreciation for the transparency of genomic testing, which provided insight into disease mechanisms and therapeutic rationale, and conveyed gratitude for the coordinated, compassionate medical care received.

## Methods

### Whole exome sequencing and analysis

Following informed consent and under institutional review board approval, tumor tissue and matched blood samples were collected from patients. Genomic DNA was extracted using standard protocols and assessed for quality and yield. Whole-exome sequencing (WES) libraries were constructed using the IDT xGen Exome Research Panel Version 2 with additional spike-ins, capturing coding and selected non-coding regions of the human genome (~620 kb of RefGene coding regions). Sequencing was performed at the Yale Center for Genome Analysis (YCGA) on an Illumina NovaSeq 6000 platform, generating paired-end 2 × 100 bp reads. A mean coverage of 382.3× for tumor sample and 110.6× for matched blood was achieved.

Raw sequencing reads were processed according to the Genome Analysis Toolkit (GATK) Best Practices pipeline. Briefly, reads were aligned to the human reference genome (e.g., GRCh37) using BWA-MEM (16–18), followed by duplicate marking, local realignment around indels, and base quality score recalibration. Somatic single nucleotide variants (SNVs) and small insertions/deletions (INDELs) were called using GATK Mutect2 (v4.4.0.0) (19) in tumor-normal mode with default parameters. The gnomAD database (20) was used as a germline resource to help distinguish inherited polymorphisms. Variants with allele frequencies below the estimated contamination threshold were removed using FilterMutectCalls.

Annotation of variants was performed using VEP (21) and vcfAnnotate (22), incorporating data from ClinVar (23), OMIM (24), COSMIC (25), SIFT (26, 27), and PolyPhen (28), as well as population allele frequencies from gnomAD. InterPro and gene ontology (GO) databases provided protein domain and functional context (29). A stringent, custom filtering strategy was then applied to the candidate variant list. Variants were retained if (1) they had a maximum sub-population frequency <0.01 in gnomAD, and (2) were predicted to have either “High” or “Medium” impact based on integrated variant effect prediction tools (VEP). Variants passing the GATK filter (“PASS”) required a minor allele frequency (MAF) >10%. Variants failing GATK filters were still considered if they had MAF >40% and were in genes cataloged in the Cancer Gene Census (30). Any variant annotated as “multiallelic” was excluded to avoid uncertain allelic interpretations. This approach was designed to enrich potentially pathogenic and functionally significant variants. CNVs and allelic imbalances were inferred using GATK’s CNV workflow, involving DenoiseReadCounts and ModelSegments, which generated denoised copy ratio and allele fraction profiles. The final integrated dataset combined high-confidence somatic variants with structural insights from CNV and allele fraction analyses, enabling a comprehensive genomic characterization of each tumor sample.

Germline variant calling was performed as previously described in our recent study (31). Briefly, whole exome sequencing data from blood sample was processed using the GATK HaplotypeCaller, and rare germline variants were prioritized based on population frequency filtering and ClinVar (23) pathogenicity evaluations.

## Discussion

SBP is a rare presentation of plasma cell neoplasms, often considered extramedullary given its occurrence outside the bone marrow microenvironment. While typically diagnosed in adulthood and often responsive to conventional treatments such as radiotherapy, a subset of SBP can transform into multiple myeloma (MM), thereby adopting a far more aggressive clinical course (32). Given the rarity of SBP, large-scale genomic studies are lacking, and most insights into its pathogenesis are derived from individual case reports. In this study, we present a comprehensive somatic genomic characterization of SBP to better understand its molecular landscape and identify potential therapeutic targets.

In our case, whole-exome sequencing (WES) revealed somatic mutations in *ARID1A*, *KMT2D*, *BCL7A*, and *PTPN11*, as well as *NUP214* in the presence of germline ATM mutation. These somatic alterations have been implicated in various processes central to plasma cell neoplasms, from chromatin remodeling (*ARID1A*, *KMT2D*, and *BCL7A*) to oncogenic signaling (*PTPN11*, *NUP214*). Of note, *ARID1A*, a core component of the SWI/SNF complex, is frequently inactivated in a range of malignancies, leading to widespread transcriptional dysregulation and chromosomal instability (32, 33). Although *ARID1A* alterations are most prominently described in ovarian and endometrial cancers, their identification in this skull-based lesion aligns with increasing evidence that epigenetic dysregulation may be a crucial driver of neoplastic transformation in plasmacytomas (1, 34, 35).

Parallel to *ARID1A*, the *KMT2D* mutation detected here also suggests a role for dysregulated histone methylation in SBP. In MM, *KMT2D* aberrations can impair glucocorticoid (GC)-based therapy by destabilizing the glucocorticoid receptor (GR), thereby reducing the apoptotic response to GCs (36–38). Such therapy resistance mechanisms are particularly concerning in the context of extramedullary disease, which has been associated with poorer prognoses and complex karyotypes (39, 40). Additionally, the identification of a potentially deleterious mutation in *BCL7A*—a gene also involved in the SWI/SNF complex—mirrors patterns observed in MM, where *BCL7A* aberrations can disrupt normal B-cell development and promote disease progression (41). Although no somatic LOH was detected at BCL7A, the presence of two somatic missense variants (S5P and R29C) suggests a possible role for loss of BCL7A in SBP pathogenesis, consistent with alterations reported in more advanced plasma cell neoplasms (42).

The detection of *PTPN11* (encoding SHP2) mutations highlights another notable parallel with MM and other hematologic malignancies, particularly those harboring RAS/MAPK pathway dysregulation (8, 43, 44). Activating mutations in *PTPN11* can enhance proliferative signals and confer drug resistance, posing significant therapeutic challenges. This observation is in parallel with the study by Bingham and colleagues (45), who performed whole-genome sequencing on extramedullary MM and reported frequent RAS/MAPK pathway aberrations, complex structural variants, and elevated subclonal diversity in patients exhibiting plasmacytomas outside the bone marrow. Bingham et al. also noted a high incidence of cytogenetic complexity, including gains at 1q—a finding mirrored in our patient’s extensive CNV profile. These shared features suggest that extramedullary plasma cell tumors, such as SBP, may exhibit overlapping molecular characteristics with other advanced plasma cell neoplasms, including evidence of epigenetic dysregulation and activated oncogenic signaling.

Pathogenic germline ATM mutations were identified in individuals with Ataxia-Telangiectasia (AT), with an autosomal recessive inheritance model. While individuals with heterozygous ATM mutations do not have AT, it is reported as a cancer predisposition factor, as ATM gene orchestrates double-strand break signaling (DBSs) and replication-stress responses, a hallmark of cancer (46, 47). In plasma-cell neoplasms, ATM pathway disruption has been associated with adverse cytogenetics and treatment resistance (48). The CNV landscape (~45% genome affected) and 1q amplification in this case align with an ATM-deficient phenotype.

The reported somatic mutational profile suggests potential treatment targets/pathways for exploration in this rare tumor context. ARID1A loss in SWI/SNF is linked to DNA-damage response vulnerabilities, including synthetic-lethal sensitivity to ATR inhibition and potential responsiveness to PARP inhibitors; ARID1A-mutant models have also shown susceptibility to EZH2 blockade (49). KMT2D alterations disrupt H3K4 methylation and broader enhancer programs, nominating chromatin-directed strategies aimed at restoring H3K4 methylation or modulating cooperating epigenetic regulators (50). Co-mutation in BCL7A further implicates BAF/SWI-SNF dysfunction; in MM, BCL7A loss permits IRF4-driven transcriptional rewiring, providing a rationale to test BAF/EZH2-axis targeting (1). The PTPN11 (SHP2-E76K) hotspot predicts RAS/MAPK activation and prevalent in various cancer types, leading to the active investigation of SHP2 inhibitors and downstream MEK/ERK blockade (51, 52). Finally, germline/functional ATM loss has been associated with heightened ATR-inhibitor sensitivity across models and select clinical contexts, with a potential risk of normal-tissue toxicity (53).

Our mutation profile (ARID1A, KMT2D, BCL7A, PTPN11, NUP214) and high CNV load converge with patterns reported across extramedullary/myeloma cohorts, notably MAPK pathway lesions and 1q amplifications. Compared with the sparse SBP-specific genomic literature, our case adds the co-occurrence of SWI/SNF alterations with PTPN11 in the setting of germline ATM, reinforcing a composite instability/signaling model for skull-base disease. While individual events have precedent in MM, their combination here and the skull-base location underscore the need for SBP-focused genomic aggregation.

Therapeutically, SBP has commonly been managed with radiotherapy, especially when progressive symptoms or lesion growth is noted. In line with the existing literature, our patient experienced clinical and radiological improvement following local radiation. Nevertheless, several chemotherapy regimens—often borrowing from MM treatment paradigms—have been explored for advanced or refractory cases. Bortezomib-based regimens, particularly when combined with steroids and other agents, have shown promise in treating extramedullary plasmacytomas (54–56). Additionally, surgical interventions may be considered in specific cases but can be technically challenging due to anatomical constraints at the skull base.

Our sequencing of local radiotherapy followed by Dara-VRd reflects three considerations. First, anatomical control is paramount at the skull base; definitive RT reliably achieves local response. Second, the tumor’s genomic risk features (1q gain; extensive CNA burden) and driver spectrum overlapping MM raise concern for occult or future systemic disease, favoring a proteasome inhibitor–IMiD–anti-CD38 backbone with demonstrated activity in extramedullary settings. Third, early symptom relief enabled timely systemic therapy without surgical morbidity in a high-risk location. Alternative strategies (e.g., Vd/VTD-based regimens or surgery) were considered; however, resection surgery offered limited incremental control given bony/dural involvement, and PI-only regimens may be suboptimal in genomically complex disease. Our observed clinical and radiologic responses support this sequencing while acknowledging the absence of randomized data in SBP (39, 40, 54–56).

Despite these various treatment modalities, SBP remains a challenging entity because of its rarity and potential for aggressive behavior. The fact that 50–60% of solitary bone plasmacytomas transform into MM within two years highlights the need for vigilant follow-up and underscores the importance of identifying genetic markers predictive of progression (10, 11). Bingham et al.’s comprehensive genomic analysis of extramedullary MM (45), combined with the insights gleaned from our case, emphasizes that extramedullary manifestations may be propelled by an accumulation of clonal drivers and structural alterations. Understanding these events could pave the way for earlier intervention and more personalized therapy selection, potentially preventing or delaying the transition to full-blown MM.

In conclusion, our findings provide a window into the genomic complexity of SBP, revealing shared driver mutations and pathway disruptions seen in MM and other extramedullary plasma cell dyscrasias. The identification of epigenetic regulators (*ARID1A*, *KMT2D*, *BCL7A*) and a key oncogenic signaling mediator (*PTPN11*) highlights the multifaceted nature of SBP pathogenesis. Further large-scale studies focused on SBP are necessary to elucidate the full spectrum of genetic heterogeneity, improve risk stratification, and guide the development of targeted therapies for this rare yet clinically significant neoplasm.

As the presented findings are derived from a single patient, the causality and generalizability of the observations are inherently limited. Tumor sampling from a skull base lesion may introduce regional heterogeneity and potential under-representation of subclonal populations. Although we performed deep sequencing (~300× coverage), applied stringent variant-calling filters, and manually confirmed all reported variants, low-VAF events may remain under-called. Biological and therapeutic inferences are extrapolated from published multiple myeloma and extramedullary plasmacytoma (MM/EMP) literature. These limitations notwithstanding, the integrated clinicogenomic analysis provides a transparent and hypothesis-generating foundation for future studies of skull base plasmacytomas.

## Conclusion

In this report, we have provided a detailed genomic characterization of a rare skull-base plasmacytoma in the setting of germline ATM mutation, demonstrating that integrative molecular analyses can yield crucial insights into the pathogenesis and potential vulnerabilities of these tumors. Our findings reveal somatic alterations in ARID1A, KMT2D, BCL7A, PTPN11, and NUP214 as possible drivers. Moreover, the extensive burden of copy number alterations reflects a high degree of genomic instability, a common feature across various tumor types and specifically in plasma cell tumors such as multiple myeloma. While therapeutic approaches for solitary SBP remain heterogeneous—ranging from radiotherapy to combination regimens used in multiple myeloma—our study highlights the importance of personalized genomic profiling in optimizing patient outcomes. Further investigation with larger cohorts and functional assays is warranted to better define prognostic biomarkers, refine risk stratification, and identify targeted strategies that could improve long-term disease control for patients with skull-base plasmacytomas.

## Data Availability

The datasets presented in this study can be found in online repositories. The names of the repository/repositories and accession number(s) can be found below: https://www.ebi.ac.uk/ena, PRJEB89814.
